# A modelling tool for capacity planning in acute and community stroke services

**DOI:** 10.1186/s12913-016-1789-4

**Published:** 2016-09-29

**Authors:** Thomas Monks, David Worthington, Michael Allen, Martin Pitt, Ken Stein, Martin A. James

**Affiliations:** 1 NIHR CLAHRC Wessex, Faculty of Health Sciences, University of Southampton, Southampton, SO17 1BJ UK; 2 Lancaster University Management School, Lancaster University, Lancaster, LA1 4YX UK; 3 NIHR CLAHRC South West Peninsula, University of Exeter Medical School, University of Exeter, Exeter, EX1 2LU UK

**Keywords:** Stroke, Capacity planning, Simulation, Average occupancy

## Abstract

**Background:**

Mathematical capacity planning methods that can take account of variations in patient complexity, admission rates and delayed discharges have long been available, but their implementation in complex pathways such as stroke care remains limited. Instead simple average based estimates are commonplace. These methods often substantially underestimate capacity requirements.

We analyse the capacity requirements for acute and community stroke services in a pathway with over 630 admissions per year. We sought to identify current capacity bottlenecks affecting patient flow, future capacity requirements in the presence of increased admissions, the impact of co-location and pooling of the acute and rehabilitation units and the impact of patient subgroups on capacity requirements. We contrast these results to the often used method of planning by average occupancy, often with arbitrary uplifts to cater for variability.

**Methods:**

We developed a discrete-event simulation model using aggregate parameter values derived from routine administrative data on over 2000 anonymised admission and discharge timestamps. The model mimicked the flow of stroke, high risk TIA and complex neurological patients from admission to an acute ward through to community rehab and early supported discharge, and predicted the probability of admission delays.

**Results:**

An increase from 10 to 14 acute beds reduces the number of patients experiencing a delay to the acute stroke unit from 1 in every 7 to 1 in 50. Co-location of the acute and rehabilitation units and pooling eight beds out of a total bed stock of 26 reduce the number of delayed acute admissions to 1 in every 29 and the number of delayed rehabilitation admissions to 1 in every 20. Planning by average occupancy would resulted in delays for one in every five patients in the acute stroke unit.

**Conclusions:**

Planning by average occupancy fails to provide appropriate reserve capacity to manage the variations seen in stroke pathways to desired service levels. An appropriate uplift from the average cannot be based simply on occupancy figures. Our method draws on long available, intuitive, but underused mathematical techniques for capacity planning. Implementation via simulation at our study hospital provided valuable decision support for planners to assess future bed numbers and organisation of the acute and rehabilitation services.

**Electronic supplementary material:**

The online version of this article (doi:10.1186/s12913-016-1789-4) contains supplementary material, which is available to authorized users.

## Background

Management of capacity in acute and community pathways is complex. To analyse these systems the mathematical sciences have developed a wide range of robust analytical methods focused on queuing and patient flow, but the uptake and implementation of these methods in routine decision making remains limited in healthcare compared to other sectors [1–3]. In the absence of these models, decision makers must make capacity planning decisions based on average occupancy of wards and, in some cases, aware of the limitations of doing so, apply arbitrary uplifts to these figures. Simulation modelling is an intuitive approach to modelling that synthesises a range of data sources to support decision making for complex problems [4]. For capacity planning problems simulation modelling offers a way to translate the large knowledge base of relevant mathematical models to a form accessible and transparent to healthcare professionals and managers.

The performance of acute and community stroke services typifies the difficulties in capacity planning decisions. Suspected stroke patients, actual stroke and mimics, require urgent access to an acute stroke unit followed by timely transfer to early support discharge services (ESD) or inpatient rehabilitation in a community hospital. Indeed in the United Kingdom the performance of stroke services is measured by the proportion of stroke patients admitted to the stroke unit within four hours of hospital arrival and the proportion of stroke patients that spend 90 % of their hospital stay on a stroke unit, with large financial penalties for underperforming services. Performance against these targets is influenced by three interacting factors [5, 6] – capacity, variation in patient length of stay and difficulties in discharging patients to the community (so called ‘bed blocking’). As the number of patients suffering a stroke increases, the pressure on acute, ESD and community rehabilitation services will rise, and accurate capacity planning that delivers a cost-effective service will become even more critical. Whilst appropriate capacity planning techniques have been implemented and used in both cardiothoracic surgery [5] and emergency departments (ED) [7], they are only outlined and encouraged with respect to stroke services [8, 9].

In any financially constrained health service there is a need for accurate capacity planning of stroke services. The present UK policy for the centralisation of hyperacute stroke services [10–13] makes it especially relevant as some stroke units will see large increases in the number of patients admitted. Capacity planning simply using average occupancy, even Bagust et al’s [14] suggested 85 % target bed occupancy, is imprecise and can lead to severe delays within the stroke pathway. Transfer delays to rehabilitation negatively affect patient outcomes [15] and may have financial penalties for hospitals. Mathematical modelling of the whole pathway provides a rational and robust way to mitigate against these problems.

### Aims

To implement advanced capacity planning techniques within a stroke pathway in a UK hospital, we developed a discrete-event simulation model based on 46 months of data (*n* = 2444; average 637 admissions per year) collected between January 2010 and October 2013. The model mimics the flow of patients from admission to an acute stroke unit through to community rehabilitation and ESD. We sought to identify current capacity bottlenecks affecting patient flow; future capacity requirements in the presence of increased admissions; the impact of co-location and pooling of the acute and rehabilitation units; and the impact of complex-neurological patients, who are also cared for on stroke wards, on capacity requirements. We contrast these results to the often used method of planning by average occupancy with and without small uplifts (10-40 %).

## Methods

### Study setting

The stroke wards in our hospital are part of a pathway that admits stroke (*n* = 1320; 54 %), high risk transient ischemic attack (TIA; *n* = 158; 6 %), complex neurological (*n* = 456; 19 %) and other types of medical patients (*n* = 510; 21 %). The acute stroke unit and single community rehab unit are in separate geographic locations. ESD is provided to mild to moderate severity stroke patients [16, 17] (*n* = 463) from both the acute (*n* = 300; 63 %) or community rehabilitation wards (*n* = 163; 37 %). The numbers of beds in the acute and rehabilitation wards are currently 10 and 12 respectively.

### Simulation model

Patient arrival rates, flows and occupancies of stroke units are subject to substantial variation due to patient type and complexity, eligibility for ESD, seasonal (daily and quarterly) effects, and overflow from other pressured hospital wards. We constructed a model incorporating these variations using the simulation software SIMUL8 [18]. The model provided a visual display of patient flows to facilitate explanation of its logic to clinicians. The model parameters are included in the online Additional file 1.

**Fig. 1:**
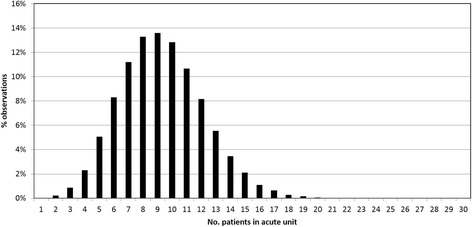
Simulation probability density function for occupancy of an acute stroke unit

**Fig. 2:**
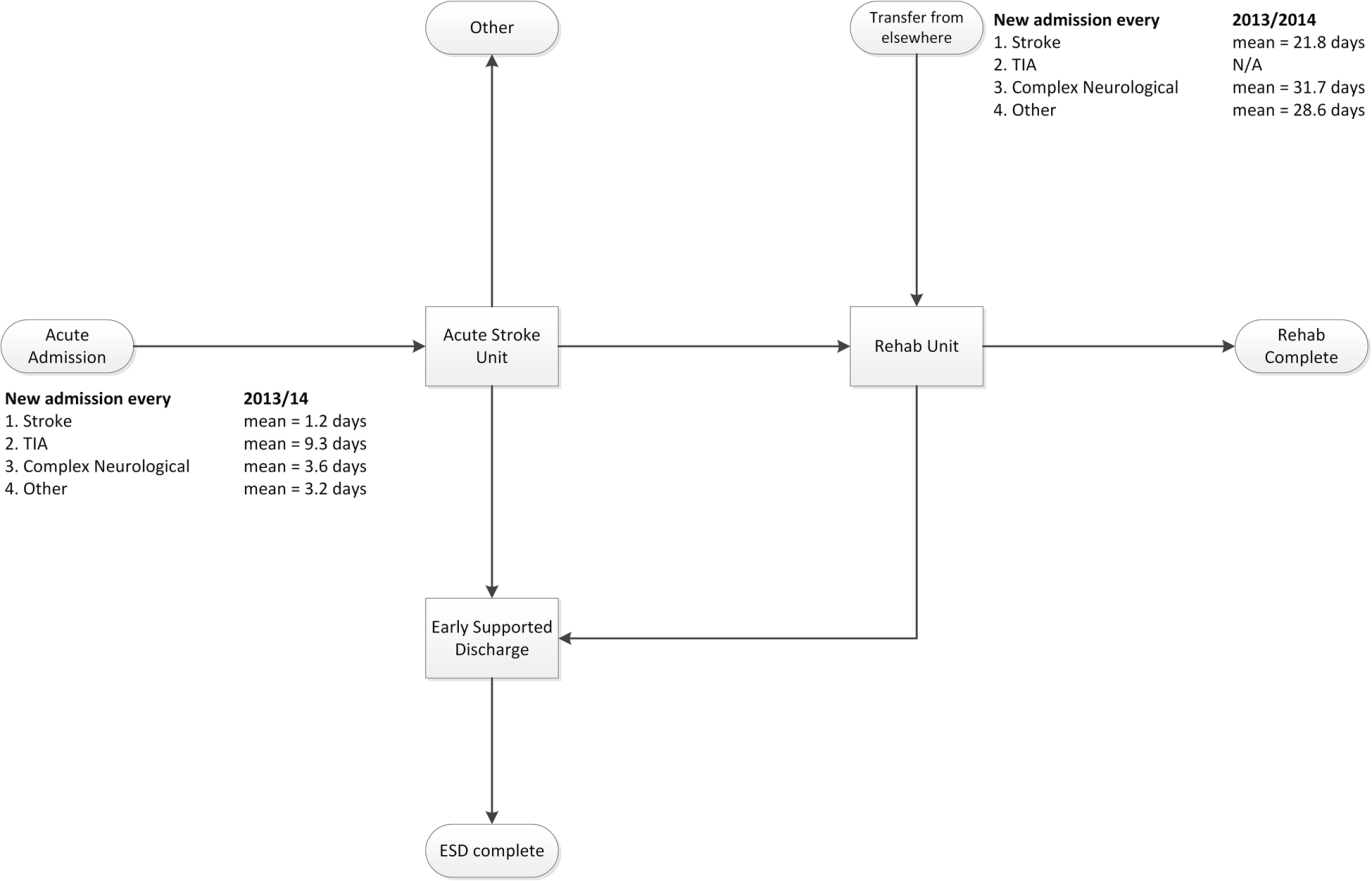
Model diagram. Notes: the arrows illustrate the destinations that patients can flow in the model. Figures are average time *between* required admissions. E.g. a stroke patient requires a bed in the acute stroke unit every 1.2 days

Our model differs from other models of stroke services focusing on thrombolysis [19–26], as it aims to inform decisions on capacity planning in different parts of the system. The key premise of our model that suits its use in capacity planning is that, unlike the real world, it allows patients to flow to the appropriate ward as soon as that is required, thereby estimating ‘unfettered’ demand [27]. The model produces a daily audit of the occupancy of each stroke ward or service and over time constructs the occupancy probability distribution function (PDF). As the model has no capacity limits, daily occupancy is Poisson distributed [28]. Figure 1 illustrates a simulated occupancy distribution with an average of nine beds, along with a clear indication of the variability away from that average. Figure 2 illustrates the model’s structure and the average admission rates of patient subgroups to the stroke wards.

### Outcome measures

**Fig. 3:**
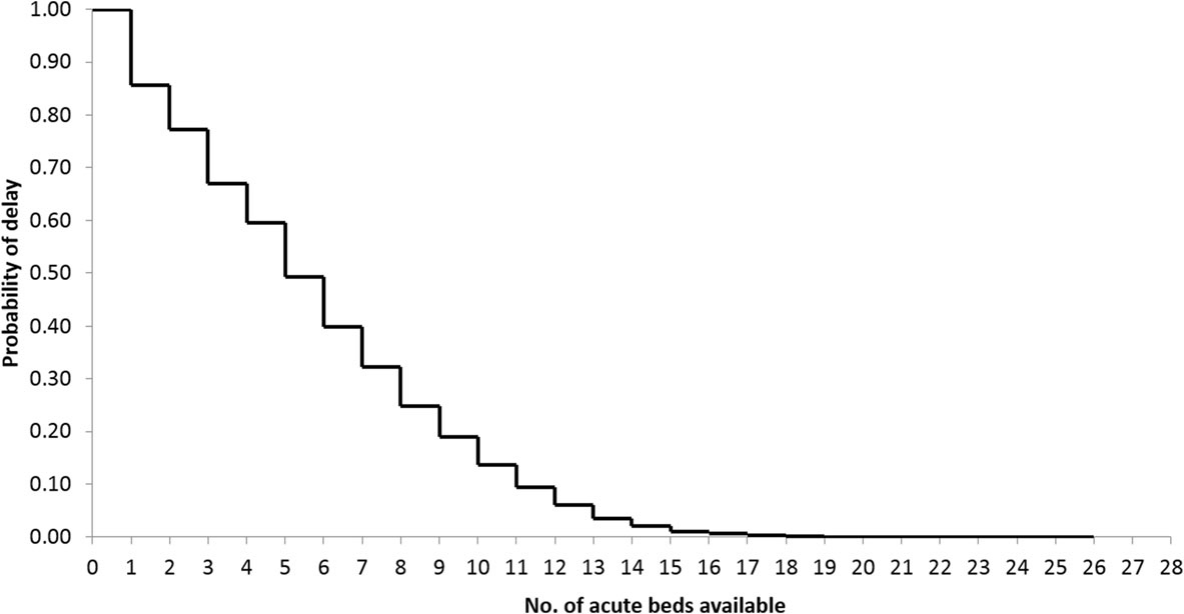
Simulated trade-off between the probability that a patient is delayed and the no. of acute beds available

The model estimates the probability that a patient cannot be immediately admitted to the acute unit, community rehabilitation unit or ESD. We call this estimate the *probability of delay* or for shorthand *p(delay)*. For each scenario investigated we estimate *p(delay)* for a range of bed numbers and construct a stepped trade-off curve (see Fig. 3 for an example). The reciprocal (1/*p(delay)*) provides a quantity that is easily understood by clinicians and managers. For example, *p(delay)* = 0.02 means that 1 in every 50 patients will experience some delay in admission or transfer.

We use both the PDF and cumulative probability density function of occupancy to calculate the probability of delay. The general form of this calculation, often referred to as the Erlang loss formula [28], is *P*(*N* = *n*)/*P*(*N* ≤ n). The calculation of the probability of delay in a system where beds are partially pooled between different types of patient is detailed in the Additional file 1.

### Data sources

The model was constructed using anonymised administrative data collected routinely by the healthcare provider in the acute and community settings. All patients had a recorded primary diagnosis using ICD-10 coding. These codes were grouped into a simpler coding scheme of stroke (ischemic or haemorrhagic), TIA, complex neurological and other. The ‘other’ category represents medical patients who are displaced into the stroke units due to capacity constraints elsewhere in the hospital.

### Statistical analysis

Variations in arrival of new admissions and length of stay are modelled using probability distributions. Exponential distributions are used to model the time between arrivals of new admissions while lognormal distributions are used to model length of stay. Each of the four patient types included in the model had their own admission and length of stay distributions, which also depended on the ward and on and the patient’s eligibility for ESD. We assumed no significant correlation between the length of a patient’s acute stay and rehabilitation stay. No data were available for length of stay in ESD. The model therefore estimates capacity requirements for acute and rehabilitation beds only.

### Scenario comparison

Table 1 lists the five scenarios used for capacity planning. To obtain stable results each scenario has a run length of five years and was replicated 150 times. As our model starts with no patients occupying beds, we also include an additional 3 year warm-up period to allow the model to reach realistic and steady-state occupancy levels. This is removed before conducting our analysis to eliminate the bias caused by the unrealistic starting state.

**Table 1:** Scenarios used for capacity planning

Scenario	Description
0. Current admissions	Current admission levels; beds are reserved for either acute or rehab patients
1. 5 % more admissions	A 5 % increase in admissions across all patient subgroups.
2. Pooling of acute and rehab beds	The acute and rehab wards are co-located at same site. Beds are pooled and can be used by either acute or rehabilitation patients. Pooling of the total bed stock of 22 is compared to the pooling of an increased bed stock of 26.
3. Partial pooling of acute and rehab beds	The acute and rehab wards are co-located at same site. A subset of the 26 beds are pooled and can be used by either acute or rehab patients.
4. No complex-neurological cases	Complex neurological patients are excluded from the pathway in order to assess their impact on bed requirements

### Model verification and validation

Input data representing patient classification into stroke and other conditions were coded and checked separately by a clinician and a data analyst working on the project. Data representing arrival rates, length of stay, and patient routing were screened and analysed by the authors and then reviewed by experienced stroke pathway staff.

To estimate arrival and length of stay distributions we followed standard practice in discrete-event simulation studies (see [29, 30]). Inter-arrival times were modelled using the exponential distribution, implying random arrivals. For length of stay we used the software Stat::Fit [31] to provide a list of candidate distributions and maximum likelihood estimates of parameters. We selected the log-normal distribution from this list as it is often used to model process times [29].

A workshop was held to review the model logic. Face validation was sought from those that worked in the stroke pathway; in this case a senior ED medic, a senior stroke physician, a senior stroke nurse, the stroke pathway manager and the hospital’ s data analyst for stroke. Explanation of the model logic was aided by an animation of the model illustrating the flow of patients. The workshop also provided a forum to review data used in the model. Initial runs of the model with parameter settings matching recent data gave model predictions consistent with recent observed system performance.

The programming of the model was verified in two ways. First, standard testing approaches [32] were applied, for example extreme value tests for arrival rates for different groups of patients entering the model and for patient routing probabilities. Second, the model underwent peer review by a specialist researcher who had not been involved in programming the model.

## Results

### Current and future admissions

The scenarios for current and future admission levels with different bed capacities are summarised in Table 2 with *p(delay)* reported to two decimal places. Planning by average occupancy of the acute unit (9 beds) and rehabilitation ward (10 beds) leads one in five patients experiencing a delay in admission. The acute stroke unit currently has 10 beds (average occupancy plus a ~10 % uplift) with a *p(delay)* of 0.19 (one in every seven patients). Even with a ~30 % uplift on average occupancy (12 beds) it is expected that one in every 16 patients experience a delay. If the number of acute beds is increased from 10 to 14 (56 % uplift) then *p(delay)* falls from 0.14 to 0.02 (1 in every 50 patients), with diminishing returns for each extra bed.

**Table 2:** Likelihood of delay. Current admissions versus 5 % more admissions

	Current admissions		5 % more admissions	
No. acute beds	p(delay)^a^	1 in every n patients delayed	p(delay)	1 in every n patients delayed
*9* ^*b*^	*0.19*	*5*		
10	0.14	7	0.16	6
11	0.09	11	0.11	9
12	0.06	16	0.07	13
13	0.04	28	0.05	21
14	0.02	50	0.03	34
No. rehab beds				
*10* ^*b*^	*0.20*	*5*		
12	0.11	9	0.13	8
13	0.08	13	0.09	11
14	0.05	20	0.07	15
15	0.03	35	0.04	25
16	0.02	57	0.02	42

^*a*^ *P(delay)* shown to 2 decimal places only

^*b*^Average occupancy with current admissions rounded to nearest number of beds

The 12 bedded rehabilitation ward represents a 20 % uplift on average occupancy. Transfers and admissions to rehabilitation have a *p(delay)* of 0.11 (one in every nine patients). An increase in rehabilitation beds to 14 (average occupancy plus a 40 % uplift) would reduce *p(delay*) to 0.05 (1 in every 20 patients). A total of 16 rehabilitation beds (60 % uplift) are required to achieve a similar *p(delay)* to 14 acute beds.

An increase of admissions by 5 % in a 14 bed acute stroke unit increases *p(delay)* from 0.02 to 0.03 (1 in every 34 patients). A 14 bed rehabilitation unit would experience an increase from 0.05 to 0.07 (1 in every 14 patients) while the operation of a 16 bed rehabilitation unit would be relatively unaffected.

### Co-location and bed pooling

We considered two pooling scenarios where the acute and rehabilitation units are co-located. The first is complete pooling of the current stock of 22 beds. In the second we consider the impact of an additional four beds and the impact of complete pooling versus pooling of a subset of the 26 beds.

Full pooling of the current bed stock reduces *p(delay)* for both acute and rehabilitation patients to 0.06 (1 in 18 patients). If an additional four beds were available and pooled the likelihood of delays drops to 1 in 64 patients. Table 3 reports this result along with results from scenarios where the units are co-located, but only a subset of the 26 beds are pooled (range 0 to 9 beds). This demonstrates that pooling can be beneficial, but that there is also a trade-off between acute delays and rehabilitation delays. As more beds are pooled this trade-off diminishes.

**Table 3:** Results of pooling of acute and rehab beds

No. beds			P(delay)^a^		1 in every n patients delays	
Dedicated Acute	Dedicated Rehab	Pooled	Acute	Rehab	Acute	Rehab
0	0	22	0.057	0.057	18	18
0	0	26	0.016	0.016	64	64
14	12	0	0.020	0.117	50	9
11	11	4	0.031	0.077	29	13
11	10	5	0.027	0.080	37	12
10	10	6	0.033	0.057	30	17
10	9	7	0.030	0.060	34	17
9	9	8	0.035	0.049	29	20
9	8	9	0.034	0.051	30	20

^a^ *P(delay)* shown to 3 decimal places

### Effect of complex neurological patients on flow

The final scenario analyses the impact of the complex-neurological patients on delays in the stroke pathway. Our hospital manages all complex-neurological patients in the acute stoke unit (some admitted as suspected stroke) for a short time; however, 11 % of complex-neurological patients are later transferred and managed in the community rehabilitation unit. These transferred patients have an effect on the delays experienced accessing rehabilitation in a 12 bed unit: increasing the number experiencing delay from 1 in every 17 patients to 1 in every 9. An effect is also seen in the acute stroke with 10 beds with the number experiencing delay increasing from 1 in every 11 patients to 1 in every 7. To achieve a 0.02 probability of a patient experiencing a delay entering the acute stroke unit 14 beds are needed with complex-neurological patients included and 13 without. A full table of results is provided in the Additional file 1.

## Discussion

We emphasise that our model’s utility is in *capacity planning* and in particular understanding the trade-off in the chance of delays under different capacity scenarios. By design the model is a simplification of the real world as it allows patients to flow to where they need to go, and hence estimates ‘unfettered’ demand. This simplification is at the heart of the models usefulness: it allows users to understand the actual capacity requirements in different parts of the pathway.

At our study hospital the model demonstrates that an increase from 10 to 14 acute stroke unit beds reduces the number of patients experiencing delays from 1 in every 7 patients to 1 in every 50. This is a substantial improvement in smoothing the flow of patients through the stroke unit and significantly increases the time clinicians can focus on patient care as opposed to bed management. Moreover, the model demonstrates that the additional four beds is relatively robust to a 5 % increase in admissions. The modelling also predicts a capacity shortfall in the inpatient rehabilitation wards. An increase from 12 to at least 14 beds is again required to smooth the flow and reduce the likelihood of transfer delays. Obvious extensions to the study are to use the model to explore the impact of reductions in rehabilitation length of stay that could result from improved discharge planning; reduction in the time to set up a community care package (reductions in ‘bed blocking’); or extending the capacity of ESD services to care for more severely affected patients – potential greatly reducing length of stay [16].

The study hospital was also planning to co-locate the acute stroke unit and rehabilitation wards. Even if bed pooling between the two units is not officially sanctioned, in practice it is likely that some temporary bed pooling will happen in order to cope with the spontaneous variation in rates of patient admissions and discharges. The model therefore provides a prospective way to plan the implementation of bed pooling and to fully understand the trade-offs when pooling only a subset of beds.

The model was also used to analyse the impact of complex-neurological patients on flow through the pathway. The utility of such information is in the dialog between clinicians and healthcare commissioners to understand the implications of service provision to different patient subgroups on overall performance.

There are several further ways in which our model can be used, depending on the issues seen to be important in different contexts. For example, it could be used to explore scenarios where stroke beds are reserved exclusively for patients suffering an acute stroke (so called ‘ring-fencing’), or ‘partial ring-fencing’ in which admissions of other cases is dependent on ward occupancy. The unfettered demand approach used in our model is generalizable and hence is applicable to other relevant wards. For example, a second use for our model would be to adapt it for other hospital wards, such as those for the cardiac surgery, where timely admission and discharge are important.

The strengths of our approach to capacity planning are threefold. First, the model provides a sophisticated analysis of capacity requirements accounting for the spontaneous and unpredictable variability in patient arrivals and lengths of stay. This level of detail is often missing from capacity calculations. Planning models that rely on average occupancy only will greatly underestimate bed requirements as they take insufficient account of variability. In this study average occupancy of the 10-bedded acute stroke unit was nine patients, corresponding to delays for one in every five patients. Our study provides a scientific methodology for analysing how many beds above average occupancy are necessary in order to limit the probability of delay. Second, although sophisticated, the model is driven by routinely collected data that is readily available from patient administration systems. Last, as the planning model has no capacity constraints, it is not necessary to model what happens to patients when stroke wards are full. Its independence of these details, which can vary considerably across hospitals, greatly increases the applicability of the model to other settings.

When adapting our model for similar studies, modellers may face the issue of dealing with the impact of ‘bed blocking’ increasing the lengths of stay recorded in routinely collected data. That is, the length of stay data do not separate treatment duration and transfer/discharge delays. If sensitivity analyses show that these discrepancies are likely to cause misleading results, a small prospective sample of times where patients are fit for transfer to rehabilitation versus when they are transferred, or a historic sample of lengths of stay during periods of time when beds are not blocked can be used.

As our model focuses on capacity requirements, a limitation is that it cannot predict the length of a delay that a patient experiences. This means that the model cannot be used to investigate performance metrics such as the UK’s four hour stroke unit target or the proportion of patients that spend 90 % of their stay on a stroke unit. Although creation of such models is possible the complexity increases by several orders of magnitude and will inevitably require data that is not routinely collected – for example regarding the management and repatriation of outlying stroke patients. The exclusion of such measures not only reduces our model’s data requirements, but also makes our approach more general internationally (where targets such as the 90 % stay metric do not apply). The model is easily adaptable to other acute stroke units which transfer patients to multiple inpatient rehabilitation wards in the community and could be used to explore the impact of introducing new cost effective services such as ESD [33].

The simulation-based method used here was chosen in preference to attempting to derive heuristics based on queueing theory for calculating the uplifts to associate with different occupancy levels as a more direct way to incorporate the characteristics of the particular problem. However, the simulation model development was guided by a knowledge of relevant queueing theories, in the spirit of complementary use of simulation and queueing theory [34].

## Conclusions

Planning by average occupancy plus an arbitrary uplift, even up to 30-40 %, fails to provide sufficient reserve capacity to adequately manage the variation in admission and discharges seen in our stroke pathway. Our method draws on long available, intuitive, but underused mathematical techniques for capacity planning. Implementation via simulation at our study hospital provided valuable decision support for planners to assess future bed numbers and organisation of the acute and rehabilitation services.

In recent years some aspects of stroke services have been modelled using discrete-event simulation approaches, [8, 19–25] including access to time-sensitive treatments such as thrombolysis. Our method, with its focus on capacity, is complementary to these models and will be particularly useful for cases of stroke service reconfigurations where acute stroke units will face substantially increased admissions, including patients for whom the final diagnosis is not stroke. To enable cost-effective and efficient provision planning decisions in such complex systems requires all of the relevant information to be considered in a way that is not possible for simple average-based estimates. Our method accounts for the variation in admission patterns, length of stay by patient type and eligibility for ESD, greatly increasing the precision with which services can be planned and the ability to predict and respond to short and long-term variation in demand for emergency stroke services.

## Additional file

Additional file 1: Supplementary methodology and results. (DOCX 28 kb)

## Abbreviations

ED: Emergency department
ESD: Early supported discharge
p(delay): The probability of a delay
PDF: Probability density function
TIA: Transient Ischemic Attack
