# Supplementary material for: A modelling tool for capacity planning in acute and community stroke services
Source: BMC Health Serv Res. 2016 Sep 29;16:530. doi: 10.1186/s12913-016-1789-4 (PMC5043535; doi:10.1186/s12913-016-1789-4)
Supplement: Additional file 1: — Supplementary methodology and results. (DOCX 28 kb) [file 12913_2016_1789_MOESM1_ESM.docx]

**A modelling tool for capacity planning in acute and community stroke services**

Monks T^a^, Worthington D^b^, Allen M^c^, Pitt M^c^, Stein K^c^, and James M^c^.

^a^NIHR CLAHRC Wessex

Faculty of Health Sciences

University of Southampton

Southampton

SO17 1BJ

UK

[thomas.monks@soton.ac.uk](mailto:thomas.monks@soton.ac.uk)

^b^Lancaster University Management School

Lancaster University

Lancaster

LA1 4YX

UK

[d.worthington@lancaster.ac.uk](mailto:d.worthington@lancaster.ac.uk)

^c^NIHR CLAHRC South West Peninsula

University of Exeter Medical School

University of Exeter

Exeter

EX1 2LU

UK

[m.allen@exeter.ac.uk](mailto:m.allen@exeter.ac.uk)

[m.pitt@exeter.ac.uk](mailto:m.pitt@exeter.ac.uk)

[Ken.Stein@exeter.ac.uk](mailto:Ken.Stein@exeter.ac.uk)

[martinjames@nhs.net](mailto:martinjames@nhs.net)

Corresponding Author

Email: thomas.monks@soton.ac.uk

Phone: 023 8120 8201

# Supplementary Methods

## Methodology for partial bed pooling

In order to conduct an analysis of partial bed pooling we used concepts from standard probability theory.   For example, consider a scenario where there are 14 acute stroke unit beds and 12 rehabilitation beds.  Two of the acute unit beds are pooled, i.e. they can be used by either acute or rehab patients. Under these circumstances we can say that:

- *Only acute patients will be delayed* if the current number of acute patients is >=14 and the current number of rehab patients is <12;
- *Only rehab patients will be delayed* if the current number of rehab patients is >=14 and the current number of acute patients is <12
- *Both acute and rehab patients will be delayed* if

the current number of acute patients is >=14 and the current number of rehab patients is >=12;

or the current number of acute patients is =13 and the current number of rehab patients is >=13;

or the current number of acute patients is =12 and the current number of rehab patients is >=14.

Continuing to use the approximation that the ward occupancies are independent, standard probability rules give us that:

P(*acute delayed*) = P*(only acute delayed*)+P(*both acute and rehab delayed*)

=P(acute>=14) x P(rehab<12) + P(acute>=14) x P(rehab>=12) + P(acute=13) x P(rehab>=13) + P(acute=12) x P(rehab>=14)

And

P(*rehab delayed*) = P(*only rehab delayed*)+P(*both acute and rehab delayed*)

=P(acute<14) x P(rehab>=12) + P(acute>=14) x P(rehab>=12) + P(acute=13) x P(rehab>=13) + P(acute=12) x P(rehab>=14)

All of these individual probabilities are provided by the simulation model.

## Supplementary Model parameters

This supplementary appendix provides the details of model parameters. These parameters replicate the base scenario, i.e. with current levels of demand. Scenarios investigating increased demand multiply the mean arrivals rates (supplied in main text) by the appropriate factor. To exclude a particular patient group the mean inter-arrival time for that group is multiplied by a large number such that no arrivals will occur in the modelled time horizon.

| **Table S2: Acute Length of stay parameters** | | | | | | | |
| --- | --- | --- | --- | --- | --- | --- | --- |
|  |  |  |  | **Percentiles** | | | |
|  | **Mean** | **Stdev** | **Median** | **5^th^** | **95^th^** | **25^th^** | **75^th^** |
| **Strokes – No ESD** | 7.4 | 8.6 | 4.0 | 1.0 | 23.0 | 2.0 | 9.0 |
| **Strokes – ESD** | 4.6 | 4.8 | 3.0 | 1.0 | 11.0 | 2.0 | 6.0 |
| **Stroke – Mortality** | 7.0 | 8.7 | 4.0 | 0.5 | 22.0 | 2.0 | 8.0 |
| **TIA** | 1.8 | 2.3 | 1.0 | 0.5 | 4.0 | 1.0 | 2.0 |
| **Complex-neurological** | 4.0 | 5.0 | 2.0 | 0.5 | 13.6 | 1.0 | 5.0 |
| **Other** | 3.8 | 5.2 | 2.0 | 0.5 | 12.1 | 1.0 | 5.0 |
| All distributions modelled as lognormal. | | | | | | | |

| **Table S2: Rehabilitation length of stay parameters** | | | | | | | |
| --- | --- | --- | --- | --- | --- | --- | --- |
|  |  |  |  | **Percentiles** | | | |
|  | **Mean** | **Stdev** | **Median** | **5^th^** | **95^th^** | **25^th^** | **75^th^** |
| **Strokes - No ESD** | 28.4 | 27.2 | 20.0 | 3.0 | 86.9 | 9.0 | 38.0 |
| **Strokes - ESD** | 30.3 | 23.1 | 22.0 | 6.0 | 78.0 | 13.8 | 44.0 |
| **Complex-neurological** | 27.6 | 28.4 | 18.0 | 2.5 | 88.5 | 8.0 | 36.0 |
| **Other** | 16.1 | 14.1 | 11.5 | 1.0 | 43.0 | 5.8 | 24.3 |
| **TIA** | 18.7 | 23.5 | 11.0 | 1.1 | 41.6 | 5.5 | 28.0 |
| All distributions modelled as lognormal. | | | | | | | |

| **Table S3: Patient transfer matrix from acute stroke unit** | | | | |
| --- | --- | --- | --- | --- |
| **Destination** | **Stroke** | **TIA** | **Complex-neurological** | **Other** |
| **Rehab** | 24% | 1% | 11% | 5% |
| **ESD** | 13% | 1% | 5% | 10% |
| **Other^*^** | 63% | 98% | 84% | 85% |
| *Other includes any destination other than rehab or ESD. For example own home, care home or mortality. | | | | |

| **Table S4: Patient transfer matrix from inpatient rehabilitation ward** | | | | |
| --- | --- | --- | --- | --- |
|  | **Stroke** | **TIA** | **Complex-neurological** | **Other** |
| ESD | 40% | 0% | 9% | 13% |
| Other | 60% | 100% | 91% | 88% |
| *Other includes any destination other than ESD. This will include the small proportion of patients that ‘bounce back’ to the acute ward. | | | | |

# Supplementary Results

## Effect of complex neurological patients on flow

| Table 2: Likelihood of delay. Current admissions versus No Complex neurological patients | | | | |
| --- | --- | --- | --- | --- |
|  | **Current admissions** | | **No Complex neurological** | |
| **No. acute beds** | **p(delay)*** | **1 in every n patients delayed** | **p(delay)*** | **1 in every n patients delayed** |
| 10 | 0.14 | 7 | 0.09 | 11 |
| 11 | 0.09 | 11 | 0.05 | 18 |
| 12 | 0.06 | 16 | 0.03 | 32 |
| 13 | 0.04 | 28 | 0.02 | 55 |
| 14 | 0.02 | 50 | 0.01 | 100 |
| 15 | 0.01 | 90 | 0.01 | 95 |
|  |  |  |  |  |
| **No. rehab beds** |  |  |  |  |
| 12 | 0.11 | 9 | 0.03 | 17 |
| 13 | 0.08 | 13 | 0.02 | 30 |
| 14 | 0.05 | 20 | 0.01 | 56 |
| 15 | 0.03 | 33 | 0.01 | 110 |
| 16 | 0.02 | 50 | 0.00 | 214 |
| *shown to 2 decimal places | | | | |

## Effect of ring fencing stroke beds on flow

| Table 3: Likelihood of delay. Current admissions versus ring fenced acute stroke beds | | | | |
| --- | --- | --- | --- | --- |
|  | **Current admissions** | | **Ring fenced acute beds** | |
| **No. acute beds** | **p(delay)*** | **1 in every n patients delayed** | **p(delay)*** | **1 in every n patients delayed** |
| 10 | 0.14 | 7 | 0.08 | 12 |
| 11 | 0.09 | 11 | 0.05 | 19 |
| 12 | 0.06 | 16 | 0.03 | 32 |
| 13 | 0.04 | 28 | 0.02 | 57 |
| 14 | 0.02 | 50 | 0.01 | 113 |
| 15 | 0.01 | 90 | 0.00 | 240 |
|  |  |  |  |  |
| *shown to 2 decimal places | | | | |
